# Increased Oxidation as an Additional Mechanism Underlying Reduced Clot Permeability and Impaired Fibrinolysis in Type 2 Diabetes

**DOI:** 10.1155/2015/456189

**Published:** 2015-08-18

**Authors:** Anna Lados-Krupa, Malgorzata Konieczynska, Artur Chmiel, Anetta Undas

**Affiliations:** ^1^Beskid Oncology Center, John Paul II Hospital, Bielsko-Biala, Poland; ^2^Department for Diagnosis, John Paul II Hospital, Krakow, Poland; ^3^Department of Cardiology, Provincial Hospital, Rybnik, Poland; ^4^Institute of Cardiology, Jagiellonian University Medical College, Krakow, Poland

## Abstract

*Aims*. We sought to investigate whether enhanced oxidation contributes to unfavorable fibrin clot properties in patients with diabetes. *Methods*. We assessed plasma fibrin clot permeation (*K*_*s*_, a measure of the pore size in fibrin networks) and clot lysis time induced by recombinant tissue plasminogen activator (CLT) in 163 consecutive type 2 diabetic patients (92 men and 71 women) aged 65 ± 8.8 years with a mean glycated hemoglobin (HbA1c) of 6.8%. We also measured oxidative stress markers, including nitrotyrosine, the soluble form of receptor for advanced glycation end products (sRAGE), 8-iso-prostaglandin F_2*α*_ (8-iso-PGF_2*α*_), oxidized low-density lipoprotein (oxLDL), and advanced glycation end products (AGE). *Results*. There were inverse correlations between *K*_*s*_ and nitrotyrosine, sRAGE, 8-iso-PGF_2*α*_, and oxLDL. CLT showed a positive correlation with oxLDL and nitrotyrosine but not with other oxidation markers. All these associations remained significant for *K*_*s*_ after adjustment for fibrinogen, disease duration, and HbA1c (all *P* < 0.05), while oxLDL was the only independent predictor of CLT. *Conclusions*. Our study shows that enhanced oxidative stress adversely affects plasma fibrin clot properties in type 2 diabetic patients, regardless of disease duration and glycemia control.

## 1. Introduction

There is compelling evidence that type 2 diabetes (T2DM) is associated with a prothrombotic state including increased thrombin generation and platelet hyperactivity as well as endothelial dysfunction [1–3]. Higher plasma D-dimer levels in diabetic patients were also observed in several studies [4].

The structure of a fibrin clot, the final step of blood coagulation, has shown to be influenced by both genetic and environmental factors [5]. A number of studies have demonstrated that the structure and function of fibrin clots are unfavorably altered in subjects with T2DM [5, 6]. Fibrin clots made from plasma of diabetic subjects have a denser, less porous structure than those formed from control plasma and they are more resistant to fibrinolysis [5]. Similar findings have been reported in studies performed on purified fibrinogen obtained from diabetic patients at various levels of metabolic control [7]. Denser fibrin networks have been shown to be related to worse glycemic control, reflected by elevated glycated hemoglobin (HbA1c). Glycation of fibrinogen may interfere with fibrin polymerization, cross-linking by FXIII, tissue plasminogen activator (tPA), plasminogen binding, and plasminogen to plasmin conversion [6]. However, it has also been demonstrated that, in some diabetic patients as compared to controls, there are no differences in clot permeability despite a marked difference in fibrinogen glycation, suggesting that other potent factors contribute to clot phenotype in this disease [7]. Of note, treatment with insulin has been shown to make fibrin more permeable despite no improvement in glycemia control [8]. Recently, it has been demonstrated that glycation of plasminogen also contributes to impaired fibrinolysis in T2DM [9].

Decreased clot susceptibility to fibrinolysis, together with high levels of plasminogen activator inhibitor-1 (PAI-1), which is typical of T2DM [10] and associated with abdominal obesity [5], may contribute to an increased atherothrombotic risk in this disease. Clinical relevance of resistance to lysis has been reinforced by observations indicating that T2DM predicts the failure of thrombolytic therapy in patients with acute myocardial infarction [11]. Recently, we have demonstrated that, even in patients with good glycemia control, prolonged duration of T2DM is associated with impaired fibrinolysis and formation of more compact fibrin clots [12].

Growing evidence indicates that enhanced oxidative stress is implicated in the pathogenesis of diabetes [13]. Elevated levels of 8-iso-prostaglandin F_2*α*_ (8-iso-PGF_2*α*_), an isoprostane that is produced by the nonenzymatic peroxidation of arachidonic acid in patients with T2DM, are detected in 24-hour urinary samples [14]. Moreover, acute glucose fluctuations were correlated with urinary excretion of 8-iso-PGF_2*α*_ [14, 15]. High serum peroxynitrite levels have been shown in diabetic patients by Al-Nimer et al. [16]. Elevated levels of circulating oxidized low-density lipoprotein (oxLDL), another oxidation marker, have also been demonstrated in patients with T2DM [17, 18]. Free radicals are formed disproportionately by glucose oxidation, nonenzymatic glycation of proteins, and the subsequent oxidative degradation of glycated proteins. High levels of free radicals and the simultaneous decline of antioxidant defense can lead to increased lipid peroxidation and insulin resistance [19]. Oxidative modifications in the fibrinogen molecules can also alter the clot structure. Fibrin deposits formed in the presence of ferric ions are remarkably resistant to proteolytic and chemical degradations. Fibrinogen modification can alter the rate of assembly of fibrin monomers into a fibrin clot and the fibrin structure [20, 21]. Associations between plasma fibrin clot properties and increased oxidative stress, reflected by plasma levels of 8-iso-PGF_2*α*_, have been reported in patients with acute myocardial infarction [22] and in heavy cigarette smokers [23]. It is unknown whether oxidative stress modifies fibrin clot characteristics in diabetes. Since T2DM markedly alters fibrin clot structure and enhances clot resistance to fibrinolysis that might predispose to cardiovascular thrombotic complications, elucidation of mechanisms underlying these abnormalities is of utmost importance. We hypothesized that increased oxidation contributes to altered plasma fibrin clot properties in T2DM.

## 2. Material and Methods

### 2.1. Subjects

We enrolled 163 consecutive white patients with T2DM diagnosed based on the WHO criteria. The exclusion criteria were as follows: signs of acute infection, known cancer, chronic inflammatory disease, liver injury (alanine and aspartate transaminase >1.5 times above the upper limit of the reference range), estimated glomerular filtration rate (eGFR) <30 mL/min, pregnancy, arterial or venous thromboembolic events within the previous 6 months, and current anticoagulant therapy, all the states known to substantially alter blood coagulation or fibrin clot structure [24].

Demographic and clinical data were collected using a standardized questionnaire. Dyslipidemia was defined as total cholesterol (TC) >5.0 mmol/L, low-density lipoprotein (LDL) cholesterol >2.6 mmol/L, and triglycerides (TG) >1.7 mmol/L or ongoing lipid-lowering treatment. Obesity was defined as the body mass index (BMI) >30 kg/m^2^. Coronary artery disease (CAD) was diagnosed based on positive results of cardiac stress test, cardiac catheterization, or coronary computed tomography angiogram. Peripheral artery disease (PAD) was defined based on the ankle-brachial index (ABI) <0.9. Previous myocardial infarction (MI), ischemic stroke, or revascularization was established based on medical records. Diabetic nephropathy was defined as macroalbuminuria (albumin to creatinine ratio [ACR] >34 mg/mmol [300 mg/g]) or microalbuminuria (ACR 3.4 to 34 mg/mmol [30–300 mg/g]) associated with retinopathy. Diabetic neuropathy was diagnosed according to the Toronto Diabetic Neuropathy Expert Group definition [25]. Diabetic retinopathy was diagnosed by an independent ophthalmologist.

The local ethics committee approved the study. All enrolled subjects provided a written informed consent.

### 2.2. Laboratory Methods

Blood was drawn after an overnight fast from an antecubital vein. Lipid profile, glucose, creatinine, blood cell, and platelet counts were assayed by routine laboratory techniques. To obtain plasma, blood samples were mixed with 3.2% sodium citrate (9 : 1) centrifuged once for 20 min and stored at −80°C. HbA1c and high sensitivity C-reactive protein (CRP) were measured using immunoturbidimetric methods (Roche Diagnostics GmbH, Mannheim, Germany). Fibrinogen was determined with the von Clauss method.

We measured using commercially available enzyme-linked immunosorbent assays (ELISA) the following oxidation markers: nitrotyrosine (Millipore, USA), advanced glycation end products (sRAGE) (R&D Systems, Minneapolis, MN, USA), serum 8-iso-PGF_2*α*_ (Cayman Chemicals, Ann Arbor, MI), and serum oxLDL (Mercodia AB, Uppsala, Sweden). To assess the advanced glycation end product (AGE) concentration, plasma fluorescence spectra were recorded with Fluoroskan Ascent FL, Labsystems (excitation 355 nm/emission 460 nm). AGE measurements were expressed in arbitral units (U/mL). This method measures a combination of glycation and oxidation products such as pentosidine, cross links, and others. Total serum fluorescence in humans is associated mainly with high molecular mass proteins, particularly albumin [26].

### 2.3. Clot Fibrin Variables

To perform fibrin clot analysis, venous blood samples were collected (vol/vol, 9 : 1) into 3.2% trisodium citrate. Plasma samples were centrifuged at 2560 g within 20 minutes of collection, immediately frozen, and stored in aliquots at −80°C until further use. Plasma fibrin clot variables were determined in duplicate by technicians not familiar with the origin of samples (intra-assay and interassay coefficients of variation, <8%), as described with slight modifications [12, 27].

Fibrin clot permeation was assessed using a pressure-driven system, with calculation of a permeation coefficient (*K*_*s*_), using the following equation:

(1) $$\begin{array}{c} K_{s}=\frac{Q\cdot L\cdot \eta }{t\cdot A\cdot \Delta p}, \end{array}$$

where *Q* is the flow rate in time *t*, *L* is the length of a fibrin gel, *η* is the viscosity of liquid (in poise), *A* is the cross-sectional area (in cm^2^), Δ*p* is a differential pressure (in dyne/cm^2^), and *t* is percolating time. Lower *K*_*s*_ values indicate reduced permeability. Briefly, 100 *μ*L of citrated plasma was mixed with 20 *μ*L of a reagent mixture at final concentrations of 10 pmol/L TF (Innovin, Siemens Healthcare Diagnostics, Marburg, Germany), 4 *μ*mol/L phospholipids (Rossix, Mölndal, Sweden) and 20 mmol/L CaCl_2_. The mixture was immediately moved to the mechanically scratched tubes. Then the gel was stabilized for 2 hours in a moisture chamber at room temperature. The tubes were connected via silicone tube to a reservoir containing Tris-buffered saline (TBS, 50 mmol/L Tris-HCl and 0.1 mol/L NaCl, pH 7.4), with a pressure drop of 4 cm H_2_O. After washing, flow rates of buffer through the clots were measured.

Clot lysis time (CLT) was measured using a tissue factor-induced clot lysis assay. Briefly, citrated plasma was mixed with calcium chloride at final concentration of 15 mmol/L, human tissue factor (Innovin, Dade Behring, Marburg, Germany) at final concentration of 0.6 pmol/L, phospholipid vesicles (Avanti Polar Lipids, Alabaster, AL) at a final concentration of 12 mmol/L, and recombinant tPA (Boehringer Ingelheim, Germany) at final concentration of 50 ng/mL. All the dilutions were prepared in Tris buffered saline (50 mmol/L Tris-HCl, 0.1 mol/L NaCl, pH 7.4). Plasma represented 50% of the mixture volume. Turbidity was measured at 405 nm at 37°C. CLT was defined as the time from the midpoint of the baseline clear to maximum turbid transition, to the final plateau phase.

### 2.4. Statistical Analysis

Statistical analysis was performed by use of MedCalc for Windows, version 13.1.2.0. All the text and tables results are expressed as mean ± standard deviation (SD) or median and interquartile range (IQR) or number and percentage. Continuous variables were compared between groups using *t*-test (variables with normal distribution) or *U* Mann-Whitney test (nonnormally distributed variables). Categorical variables were compared by use of the chi-square test. To determine the relationship between variables the Spearman rank-order or Pearson correlations were analyzed. Finally a multivariate regression model was constructed using all variables, that is, oxidative markers, duration of diabetes, HbA1c, fibrinogen, and other markers (if in univariate regression a *P* value was <0.1) in backward approach to determine which factors had influence after adjustment. A *P* value <0.05 was considered statistically significant.

## 3. Results

The final analysis included 163 patients (92 men and 71 women aged 65 ± 8.8 years, range 49–82 years) with a mean duration of diabetes of 5 years and mean HbA1c of 6.8% (Table 1). There were 127 subjects with DM duration ≤10 years and 36 with DM duration above 10 years. Vascular manifestations of T2DM were as follows: diabetic nephropathy *n* = 29 (17.8%), diabetic retinopathy *n* = 24 (14.7%), and diabetic neuropathy *n* = 33 (20.2%). One hundred patients had coronary artery disease. There were 114 (69.9%) patients with good glycemic control (HbA1c ≤7%) and 49 patients (30.1%) with inadequate glycemic control (HbA1c >7%). Forty-three (26.4%) patients were treated with insulin, and 166 patients received oral hypoglycemic drugs (biguanides and suphonylurea). There were more patients treated with insulin among those with HbA1c >7%. Subjects with HbA1c >7% like those with DM duration >10 years had higher prevalence of obesity, hypertension, dyslipidemia, CAD, previous MI, previous coronary interventions, PAD, nephropathy, and retinopathy.

### 3.1. The Relationship between Oxidation Markers

Differences in oxLDL levels investigated in plasma samples were observed between current smokers and nonsmokers (*P* = 0.04). 8-iso-PGF_2*α*_ levels were higher, while AGE levels were slightly lower, in patients with nephropathy compared with those without this complication (both *P* = 0.04). No differences were observed with regard to the presence of other concomitant diseases (Table 2).

As expected, there were positive correlations between AGE and oxLDL (*r* = 0.341, *P* < 0.0001) and AGE and nitrotyrosine (*r* = 0.244, *P* < 0.0035) as well as between sRAGE and 8-iso-PGF_2*α*_ (*r* = 0.688, *P* < 0.0001) and nitrotyrosine (*r* = 0.694, *P* < 0.0001). 8-iso-PGF_2*α*_ was also correlated with nitrotyrosine (*r* = 0.5, *P* < 0.0001) and oxLDL with nitrotyrosine (*r* = 0.218, *P* < 0.009). Fasting glucose correlated with 8-iso-PGF_2*α*_ (*P* = 0.03) but not with other oxidation parameters. In contrast, HbA1c positively correlated with nitrotyrosine (*P* = 0.006) and oxLDL (*P* = 0.02). There were no correlations between disease duration and oxidation markers (data not shown).

### 3.2. Fibrin Clot Properties and Its Relationship to Oxidation Markers

As expected, *K*_*s*_ and CLT investigated* in vitro* in plasma samples were inversely correlated in diabetic patients (*r* = −0.5, *P* < 0.0001). We found inverse correlations between *K*_*s*_ and both diabetes duration (*r* = −0.32, *P* = 0.0001) and HbA1c (*r* = −0.30, *P* = 0.0001) in the whole group. No similar associations were observed for CLT (Table 3). There were no associations between lipid profile or renal function and fibrin properties (data not shown).

Importantly, there were inverse correlations between *K*_*s*_ and all the 5 oxidation markers, that is, nitrotyrosine, sRAGE, 8-iso-PGF_2*α*_, AGE, and oxLDL (Table 3). There were also positive correlations of CLT with oxLDL and nitrotyrosine but not with other oxidation markers (Table 3).

Longer clot lysis time and lower clot permeability (after adjustment for fibrinogen and HbA1c) were observed in T2DM with longer diabetes duration. In patients with diabetes duration >10 years we observed prolonged CLT (mean value 100 min, 95% CI: 94.5–105.5) compared with the values obtained for subjects with shorter history of diabetes (mean value 93.7 min, 95% CI: 90.8–96.6, *P* = 0.048). Similarly longer diabetes duration was associated with lower *K*_*s*_ (mean value 6.8 × 10^−9^ cm^2^, 95% CI: 6.5–7.0) compared with the patients who were diagnosed with T2DM 9 years earlier or less (mean value 7.2 × 10^−9^ cm^2^, 95% CI: 7.1–7.3, *P* = 0.007).

*K*_*s*_ and CLT adjusted for fibrinogen and diabetes duration did not differ between patients with HbA1c >7% and those with HbA1c ≤7%. In patients with inadequate glucose control (HbA1c > 7%) we observed similar CLT values compared to the remainder (mean, 95.9 min, 95% CI: 91.0–100.7 versus 94.7 min, 95% CI: 91.5–97.9; *P* = 0.69, resp.).

For clot permeability differences between the two groups were also insignificant (mean, 6.9 × 10^−9^ cm^2^, 95% CI: 6.7–7.2, versus 7.2 × 10^−9^ cm^2^, 95% CI: 7.0–7.3; *P* = 0.16, resp.).

Multivariate regression analysis was performed to determine independent predictors of clot fibrin variables. Analysis adjusted for fibrinogen, disease duration, and HbA1c showed that independent predictors of *K*_*s*_ in diabetic patients were nitrotyrosine (*P* < 0.001), sRAGE (*P* = 0.001), 8-iso-PGF_2*α*_ (*P* = 0.003), and oxLDL (*P* = 0.01) (Table 4), while oxLDL (*P* < 0.0001) was the only independent predictor of CLT (Table 5).

## 4. Discussion

This study is the first to show that enhanced oxidative stress reflected by its circulating markers including nitrotyrosine, sRAGE, 8-iso-PGF_2*α*_, and oxLDL unfavorably affects the fibrin network structure and susceptibility to lysis in T2DM. Known factors contributing to the prothrombotic fibrin clot phenotype observed in patients with diabetes represent increased plasma fibrinogen concentrations and enhanced glycation of the fibrinogen or plasminogen molecules [5–9, 28]. Our observations suggest a novel prothrombotic mechanism mediated by oxidative stress that may lead to the formation of denser and resistant to lysis plasma fibrin meshwork in T2DM and thus enhancing unfavorable effects of protein glycation. Moreover, our findings provide additional evidence that oxidation can modify the clot structure.* In vitro* experiments demonstrated that the extent of oxidation and substances used to its generation strongly affect alterations to fibrin clot properties and these effects range widely from markedly impaired fibrin formation to tendency to produce compact networks [20]. We found that oxidation superimposed on the glycation in diabetes makes fibrin clots less permeable and poorly lysable.

To assess the impact of oxidation* in vivo*, we have chosen five parameters detectable in circulating blood that have been used in several studies performed in T2DM patients [29, 30]. To our knowledge, associations of these oxidation markers with fibrin clot characteristics have not been studied yet. We observed the heterogeneity of the effects of circulating oxidation markers on fibrin variables in our experimental approach, which supports the concept that complex oxidative processes occurring* in vivo* may alter fibrin clot structure and function in a different manner.

Measurement of 8-iso-PGF_2*α*_ is a reliable tool for the identification of subjects with enhanced rates of lipid peroxidation. This prostaglandin is produced by the nonenzymatic peroxidation of arachidonic acid in membrane phospholipids [19, 31]. Of special interest are our findings regarding nitrotyrosine, a product of peroxynitrite action, in the plasma of diabetic patients. A direct correlation between postprandial hyperglycemia and the production of nitrotyrosine was observed in T2DM [32]. There is evidence that peroxynitrite is generated in diabetes and might be involved in the development of diabetes complications [33].

We observed that in T2DM patients nitrotyrosine could be a good predictor of *K*_*s*_. AGEs are modifications of proteins or lipids that become nonenzymatically glycated and oxidized after contact with aldose sugars. Early glycation and oxidation processes result in the formation of Schiff bases and Amadori products. Further glycation of proteins and lipids causes molecular rearrangements that lead to the generation of AGEs [34]. Circulating AGE levels are considered a more accurate biomarker than plasma glucose or HbA1c for the assessment of cumulative glycemic control [35] and a major contributor to accelerate both the microvascular and macrovascular complications in T2DM patients [36].

Activation of RAGE, the receptor for advanced glycation end products by AGEs, causes upregulation of the transcription factor nuclear factor-*κ*B and its target genes, which control cellular expression of RAGE, linking RAGE to the inflammatory response [37]. Plasma sRAGE correlates positively with albumin excretion in T2DM patients, which may represent an early marker of diabetic nephropathy [38]. Some observations suggest the active participation of AGEs-RAGE axis in accelerated atherosclerosis and development of diabetic cardiomyopathy via inducing endothelial dysfunction, inflammatory, fibrotic, and proapoptotic reactions in the myocardium [39]. Kajikawa et al. reported that the ratio of AGEs to soluble form of RAGE may be a new chemical biomarker of endothelial function [40].

However, in our study we observed slight differences in circulating AGE levels in diabetic patients who had low and high levels of HbA1c. We have found that sRAGE alters fibrin-related prothrombotic mechanisms in T2DM.

A marker of the oxidative modification of LDL particles, plasma oxidized LDL (oxLDL), is elevated among individuals with abdominal fat, and it is positively associated with T2DM, fasting glucose, and HbA1c [41]. It has been demonstrated that oxLDL is an independent risk factor of future clinical events in a general population [42]. Importantly, oxLDL was found in the present study as the only independent predictor of prolonged CLT in T2DM, suggesting that oxLDL might directly affect the binding of fibrinolysis proteins to the fibrin fibers and thus impairing plasmin-mediated fibrin degradation.

Previous studies have demonstrated that oxidative stress parameters were elevated in patients with long-standing diabetes [43]. On the other hand, we have shown that prolonged diabetes duration is linked to persistent prothrombotic alterations despite good glycemia control [12].

A role of oxidative stress in maintaining the metabolic memory has also been suggested [44]. The current study expands the current knowledge by indicating that oxidative stress contributes to the fibrin-related aspects of metabolic memory. We showed that hypofibrinolysis and denser fibrin clot networks aggravated by oxidative stress have been associated with glycemia control and also with prolonged duration of diabetes. However, diabetes duration itself is the risk of thrombotic events despite good glycemia control. It is known that glucose control decreases fibrinogen glycation in subjects with T2DM. Glycated fibrinogen correlates well with HbA1c but it does not explain increased blood coagulation in long-term diabetes and good metabolic control of the disease [45]. It is known that oxidative stress is positively correlated with glycemic variability over a daily period. It should be noted that dipeptidyl peptidase-IV (DPP-4) inhibitors can influence oxidative stress and reduce HbA1c and glycemic fluctuations on a daily basis [46]. Pharmacological modulation of oxidative stress in diabetes merits further studies in the context of a prothrombotic state.

The study has several limitations. First, the size of the study population was relatively small but representative of real-life patients with T2DM. Second, we determined each variable in a single time point, and some changes in the levels during the follow-up cannot be excluded. Most HbA1c levels did not exceed 10%, so we did not evaluate patients with extremely high HbA1c. It should also be noted that duration of diabetes represents the time from diagnosis, not the actual duration of the disease. Finally, a large prospective study is needed to assess the links between the fibrin phenotype in the context of oxidative stress markers and thromboembolic events in patients with diabetes.

## 5. Conclusions

We demonstrated that prothrombotic alterations detectable in plasma fibrin clot characteristics of T2DM patients are associated not only with inadequate glycemia control and disease duration but also with increased oxidation.

Mechanisms behind complex interactions between glycation and oxidation remain to be established.

## Figures and Tables

**Table 1:** Patient characteristics (*n* = 163).

Variable	
Age (years)	65 ± 8.8
Male gender, *n* (%)	92 (55.4)
BMI, kg/m^2^	31.9 ± 5.2
WHR	0.98 (0.93–1.04)
Cardiovascular risk factors, *n* (%)	
Obesity	109 (66.9)
Current smoker	16 (9.8)
Previous smoker	82 (50.3)
Hypertension	153 (93.9)
Dyslipidemia	126 (77.3)
Family history of CAD	40 (24.5)
Medical history, *n* (%)	
CAD	100 (61.3)
Previous MI	27 (16.6)
Previous PCI/CABG	2 (1.2)
Previous stroke or TIA	11 (6.7)
PAD	22 (13.5)
Nephropathy	29 (17.8)
Retinopathy	24 (14.7)
Neuropathy	33 (20.2)
Insulin therapy	43 (26.4)
Biguanide	97 (59.5)
Sulphonylurea	69 (42.3)
Aspirin	130 (79.8)
Thienopyridine	9 (5.5)
Statin	128 (78.5)
Fibrate	7 (4.3)
Beta-blocker	131 (80.4)
ACEI	117 (71.8)
Laboratory results	
GFR, mL/min	78.2 ± 20.0
TC, mmol/L	4.3 (3.5–5.1)
LDL-C, mmol/L	2.4 (1.9–3.1)
HDL-C, mmol/L	1.3 (1.1–1.6)
TG, mmol/L	1.3 (1.0–1.9)
CRP, mg/L	2.2 (1.1–4.1)
Glucose, mmol/L	6.0 (5.1–7.3)
HbA1c, %	6.8 ± 1.036

Values are given as mean ± SD, median (interquartile range), or percentage.

*P* value was calculated using Student *t*-test when variables were normally distributed or by the Mann-Whitney *U*-test for nonnormally distributed variables.

Abbreviations: DM, type 2 diabetes; HbA1c, glycated hemoglobin; WHR, waist to hip ratio; CAD, coronary artery disease; MI, myocardial infarction; PCI, percutaneous coronary intervention; CABG, coronary artery bypass graft; TIA, transient ischemic attack; PAD, peripheral artery disease; ACEI, angiotensin-converting enzyme inhibitor; GFR, glomerular filtration rate; TC, total cholesterol; LDL-C, low-density lipoprotein cholesterol; HDL-C, high-density lipoprotein cholesterol; TG, triglycerides; CRP, C-reactive protein.

**Table 2:** Levels of oxidation markers in specific groups.

Variables	oxLDL	Nitrotyrosine	AGE	sRAGE	PGF_2*α*_
	ng/mL	nM/mL	U/mL	ng/mL	pg/mL
Obesity (+)	216 (185–250)	0.62 (0.59–0.70)	7.1 (6.1–8.4)	0.91 (0.85–0.98)	449 (415–480)
Obesity (−)	221 (180–273)	0.66 (0.60–0.69)	6.1 (7.1–8.1)	0.94 (0.86–0.99)	459 (406–485)
*P*	0.56	0.24	0.51	0.50	0.32
Current smoker (+)	180 (167–233)	0.66 (0.55–0.75)	6.6 (5.9–7.7)	0.88 (0.85–1.07)	451 (360–498)
Current smoker (−)	219 (189–263)	0.63 (0.60–0.70)	6.6 (5.9–7.7)	0.92 (0.86–0.99)	453 (417–481)
*P*	0.04	0.55	1.0	0.54	0.94
Previous smoker (+)	212 (180–250)	0.64 (0.60–0.70)	7.1 (6.0–8.4)	0.92 (0.87–0.99)	458 (418–481)
Previous smoker (−)	220 (186–260)	0.61 (0.57–0.68)	7.1 (6.3–8.3)	0.91 (0.85–0.98)	448 (410–480)
*P*	0.61	0.05	0.98	0.38	0.20
Hypertension (+)	217 (182–255)	0.63 (0.59–0.70)	7.1 (6.1–8.4)	0.92 (0.85–0.99)	453 (417–481)
Hypertension (−)	242 (193–266)	0.67 (0.61–0.70)	6.5 (5.4–7.1)	0.91 (0.83–1.04)	458 (398–479)
*P*	0.52	0.37	0.18	0.70	0.93
Dyslipidemia (+)	218 (186–258)	0.63 (0.60–0.69)	7.1 (6.1–8.4)	0.91 (0.85–0.99)	453 (418–480)
Dyslipidemia (−)	121 (180–253)	0.62 (0.55–0.70)	7.0 (6.3–7.8)	0.94 (0.88–0.98)	452 (401–482)
*P*	0.72	0.70	0.44	0.82	0.81
FH (+)	214 (184–247)	0.63 (0.58–0.70)	6.9 (6.0–8.1)	0.91 (0.85–0.97)	458 (417–478)
FH (−)	219 (184–265)	0.63 (0.60–0.70)	7.1 (6.2–8.4)	0.92 (0.86–0.99)	452 (409–481)
*P*	0.49	0.90	0.55	0.55	0.89
CAD (+)	218 (186–268)	0.63 (0.60–0.70)	7.2 (6.4–8.4)	0.92 (0.85–0.99)	457 (416–496)
CAD (−)	208 (178–250)	0.63 (0.57–0.67)	6.8 (5.9–8.1)	0.92 (0.87–0.99)	542 (413–480)
*P*	0.44	0.22	0.14	0.58	0.17
Previous MI (+)	198 (175–235)	0.68 (0.60–0.72)	7.5 (6.5–8.4)	0.92 (0.88–0.92)	457 (416–496)
Previous MI (−)	220 (190–260)	0.62 (0.59–0.69)	7.1 (6.0–8.2)	0.91 (0.85–0.99)	452 (413–480)
*P*	0.14	0.06	0.27	0.41	0.4
Prev. stroke/TIA (+)	198 (181–240)	0.63 (0.60–0.70)	7.4 (6.4–9.3)	0.92 (0.85–0.99)	451 (403–480)
Prev. stroke/TIA (−)	217 (185–260)	0.63 (0.57–0.67)	7.1 (6.1–8.2)	0.92 (0.87–0.99)	459 (429–491)
*P*	0.53	0.38	0.21	0.18	0.13
PAD (+)	197 (168–280)	0.60 (0.55–0.77)	7.0 (6.1–8.2)	0.91 (0.81–0.97)	420 (389–460)
PAD (−)	218 (189–254)	0.63 (0.60–0.70)	7.1 (6.1–8.4)	0.92 (0.86–0.99)	457 (421–481)
*P*	0.41	0.69	0.93	0.24	0.12
Nephropathy (+)	210 (179–243)	0.63 (0.60–0.71)	6.5 (5.9–7.3)	0.92 (0.90–1.01)	468 (440–501)
Nephropathy (−)	218 (186–264)	0.63 (0.59–0.70)	7.2 (6.2–8.4)	0.91 (0.85–0.99)	450 (402–478)
*P*	0.34	0.55	0.04	0.17	0.04
Retinopathy (+)	210 (185–244)	0.60 (0.60–0.70)	7.1 (6.3–7.7)	0.91 (0.83–0.99)	445 (398–481)
Retinopathy (−)	220 (184–258)	0.63 (0.59–0.70)	7.1 (6.1–8.4)	0.92 (0.86–0.99)	453 (417–481)
*P*	0.69	0.94	0.55	0.45	0.69

Data expressed as median interquartile range (IQR).

Abbreviations: oxLDL, oxidized low-density lipoprotein; AGE, advanced glycation end-product; sRAGE, the soluble form of receptor for advanced glycation end products; PGF_2*α*_, 8-iso-prostaglandin F_2*α*_; FH, family history of diabetes; CAD, coronary artery disease; MI, previous myocardial infarction; TIA, transient ischemic attack; PAD, peripheral artery disease.

**Table 3:** Univariate correlations of clot permeability (*K*_*s*_, 10^−9^ cm^2^) and clot lysis time (CLT, min).

		*K*_*s*_,	CLT	*K*_*s*_^1^	CLT^1^
HbA1c, %	*r*	−0.30	0.14		
	*P*	0.0002	0.10		

DM duration	*r*	−0.32	0.14		
	*P*	0.0001	0.09		

Nitrotyrosine, nM/mL	*r*	−0.44	0.19	−0.47	0.14
	*P*	<0.001	0.02	<0.001	0.08

AGE, U/mL	*r*	−0.20	0.107	−0.17	−0.02
	*P*	0.01	0.20	0.05	0.82

sRAGE, ng/mL	*r*	−0.27	0.13	−0.26	0.05
	*P*	0.001	0.11	0.001	0.53

PGF_2*α*_, pg/mL	*r*	−0.22	0.13	−0.25	0.11
	*P*	0.007	0.10	0.003	0.19

oxLDL, ng/mL	*r*	−0.33	0.48	−0.21	0.41
	*P*	0.0001	<0.0001	0.01	<0.0001

Glucose, mmol/L	*r*	−0.14	0.20		
	*P*	0.07	0.02		

^1^ *P* after adjustment for fibrinogen, DM duration time, and HbA1c.

Abbreviations: see Tables 1 and 2.

**Table 4:** Multivariate regression, dependent variable *K*_*s*_.

Independent variables	Coefficient	Standard error	*r*_partial_	*t*	*P*
(Constant)	11.49				
HbA1c	−0.11	0.056	−0.16	−1.90	0.059
DM duration	−0.02	0.007	−0.23	−2.77	0.007
Nitrotyrosine, nM/mL	−3.66	0.613	−0.45	−5.97	<0.0001
oxLDL, ng/mL	−0.002	0.0009	−0.18	−2.11	0.036
Fibrinogen, g/L	−0.22	0.0896	−0.21	−2.44	0.016

Method backward, multiple correlation coefficient 0.64; *P* < 0.001.

Abbreviations: see Tables 1 and 2.

**Table 5:** Multivariate regression, dependent variable CLT.

Independent variables	Coefficient	Standard error	*r*_partial_	*t*	*P*
(Constant)	41.05				
oxLDL, ng/mL	0.11	0.021	0.41	5.31	<0.001
Fibrinogen, g/L	9.10	2.03	0.36	4.47	<0.001

Method backward, multiple correlation coefficient 0.54; *P* < 0.001.

Abbreviations: see Tables 1 and 2.
